# Surgical Case of Metachronous Occurrence of Intracholecystic Papillary Neoplasm in the Remnant Cystic Duct 19 Years after Cholecystectomy

**DOI:** 10.70352/scrj.cr.24-0175

**Published:** 2025-06-24

**Authors:** Hiroki Kanno, Kenjiro Date, Yoshinao Kinjyo, Takeshi Aoyagi, Shota Yamashiro, Eishi Iwaoka, Ko Shigemura, Koichiro Shimonaga, Takao Tsukahara, Ryo Ichikawa, Hiroyuki Nakane, Kanako Kurata, Gentaro Hirokata, Yoshihiko Sadakari, Masahiko Taniguchi

**Affiliations:** 1 Department of Surgery, St. Mary’s Hospital, Kurume, Japan; 2 Department of Surgery, Kurume University School of Medicine, Kurume, Japan; 3 Department of Pathology, St. Mary’s Hospital, Kurume, Japan

**Keywords:** intracholecystic papillary neoplasm, metachronous occurrence, cystic duct

## Abstract

**INTRODUCTION:**

Intracholecystic papillary neoplasm (ICPN) is a recently identified disease characterized by papillary pre-invasive neoplasm of the gallbladder. Despite its characterization, the natural history of ICPN remains elusive. Furthermore, a few cases of metachronous ICPN in the remnant biliary system have been documented. Here, we report a surgical case involving metachronous ICPN in the remnant cystic duct 19 years post-cholecystectomy for primary ICPN.

**CASE PRESENTATION:**

A 77-year-old man presented to our hospital with general fatigue and jaundice. He had previously undergone an open cholecystectomy and lithotomy for gallbladder cancer and common bile duct stones 19 years earlier. Blood tests revealed elevated levels of hepatobiliary enzymes and tumor markers. Both computed tomography and magnetic resonance imaging indicated dilatation of the intrahepatic and common bile ducts, and an enhanced nodule was observed in the common hepatic duct. Intraductal ultrasonography identified a papillary tumor infiltrating the distal bile duct from the common hepatic duct. Brush cytology subsequently helped confirm adenocarcinoma. Consequently, the patient was diagnosed with Bismuth type 1 perihilar cholangiocarcinoma and underwent subtotal stomach-preserving pancreaticoduodenectomy. Histological examination revealed the tumor as pancreatobiliary-type ICPN associated with invasive carcinoma, which had originated in the remnant cystic duct and invaded the common hepatic duct. A retrospective review of the resected gallbladder specimens from 19 years earlier confirmed ICPN according to the current classification, establishing this as a metachronous occurrence of ICPN.

**CONCLUSIONS:**

These findings suggest that ICPN can recur as metachronous lesions in the remnant biliary system after resection of primary lesion, highlighting the necessity of sustained, long-term biliary surveillance following primary lesion resection.

## Abbreviations

CBD
: common bile duct
CHD
: common hepatic duct
ICPN
: intracholecystic papillary neoplasm
IPMN
: intrapapillary mucinous neoplasm
IPNB
: intraductal papillary neoplasm of the bile duct

## INTRODUCTION

ICPNs were first recognized in the World Health Organization classification of bile duct tumors in 2010.^1)^ They share clinicopathological similarities with pancreatic IPMN and IPNB, including intraluminal papillary growth patterns, variable dysplastic grades, and various phenotypes. Therefore, ICPN is considered a counterpart of IPMN and IPNB.^2)^ Given the recent identification and rarity of ICPN, the natural history of ICPN remains largely unknown. Although several metachronous occurrences of IPMN and IPNB following primary lesion resection have been documented,^3–6)^ a few reports have documented such occurrences in the biliary tract post-cholecystectomy for primary ICPN. In this report, we present a surgical case of metachronous ICPN in the remnant cystic duct 19 years following cholecystectomy for primary ICPN.

## CASE PRESENTATION

**Fig. 1:**
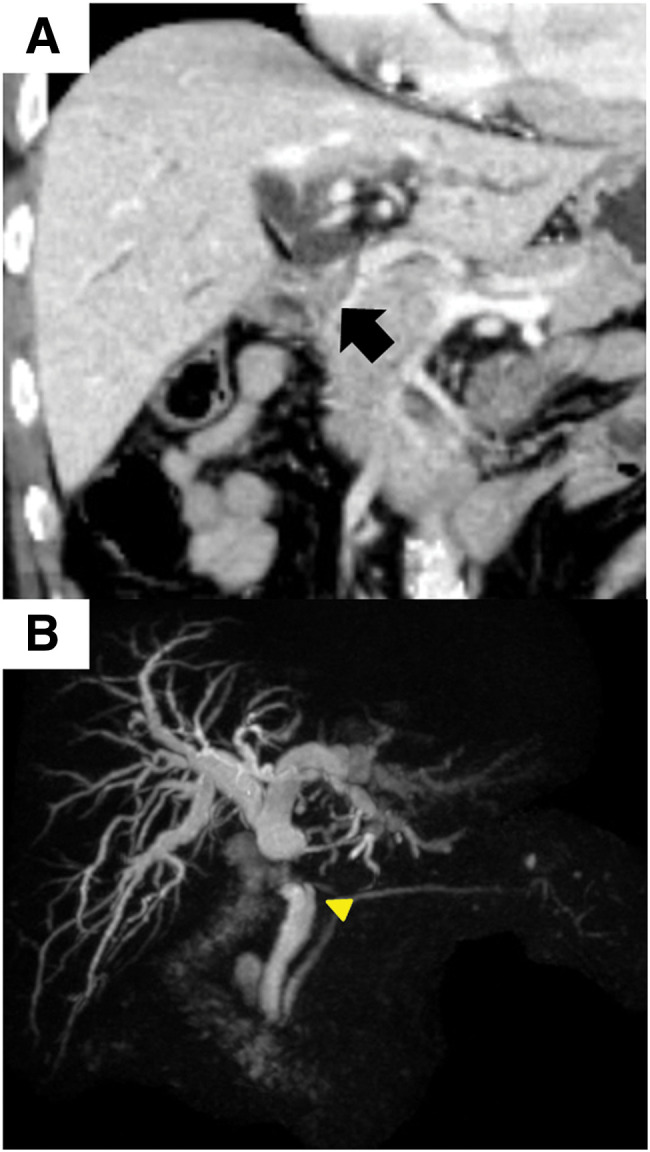
Computed tomography and magnetic resonance cholangiopancreatography findings. Computed tomography image displaying dilatation of the intrahepatic bile and common hepatic ducts. An enhanced nodule, 22 mm in diameter, is found in the confluence of the cystic duct (arrow) (**A**). Magnetic resonance cholangiopancreatography showing the defect in the common hepatic duct. Yellow arrowhead indicates the remnant cystic duct (**B**).

A 77-year-old man presented to our hospital with general fatigue and jaundice. He had a history of hyperlipidemia and was taking oral medication. Nineteen years ago, he had undergone open cholecystectomy with cystic duct lymph node dissection and lithotomy for gallbladder cancer and CBD stones. The tumor was located in the neck of the gallbladder on the hepatic side and was diagnosed as gallbladder cancer, 21 × 19 mm, G1, papillary carcinoma, non-invasive, pTis pN0 M0, pStage 0. Blood tests indicated elevated levels of hepatobiliary enzymes and tumor markers as follows: aspartate aminotransferase 231 U/L (normal reference range at our institution, 13–30 U/L), alanine aminotransferase 261 U/L (normal range, 10–42 U/L), alkaline phosphatase 1653 U/L (normal range, 38–113 U/L), gamma-glutamyltransferase 1645 U/L (normal range, 13–64 U/L), total bilirubin 4.53 mg/dL (normal range, 0.4–1.5 mg/dL), and carbohydrate antigen 19-9 at 114.0 U/mL (normal range, 0–37 U/mL). Computed tomography and magnetic resonance cholangiopancreatography revealed dilatation of the intrahepatic bile duct and CHD, with a 22-mm gradually enhancing nodule on the CHD (**Fig. 1A**, **1B**). Neither lymph node nor distant metastasis was detected.

**Fig. 2:**
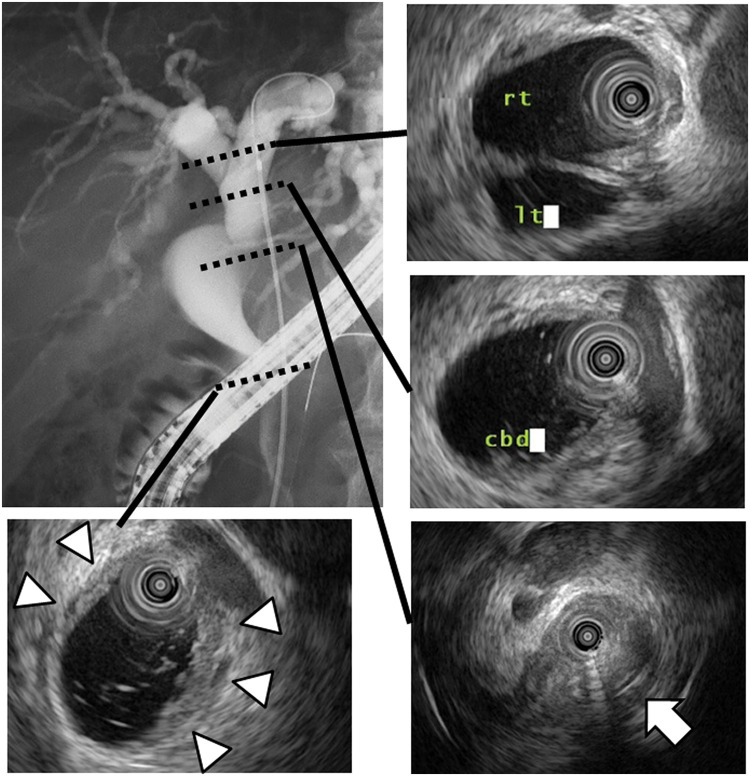
Endoscopic and ultrasonographic tumor assessments. Endoscopic retrograde cholangiopancreatography and intraductal ultrasonography displaying infiltration of the tumor from the common hepatic duct into the distal bile duct. Focal wall thickness is observed in the common hepatic duct (arrowheads), and the distal bile duct is obstructed by the tumor (arrow).

**Fig. 3:**
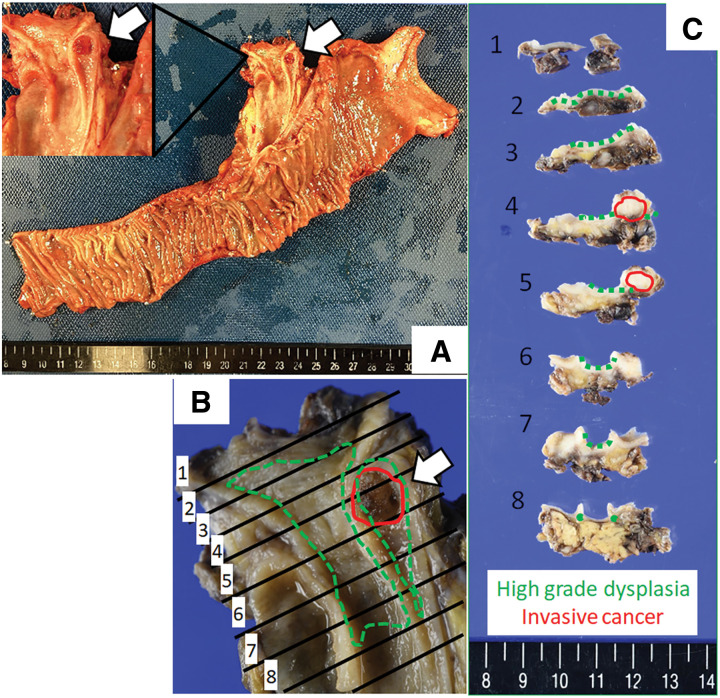
Macroscopic view of the papillary nodule. Macroscopic findings of surgical specimen (**A**). A 5-mm papillary nodule in the cystic duct is observed (arrow). Red and green dotted lines represent the areas of invasive cancer and high-grade dysplasia, respectively (**B**–**C**).

**Fig. 4:**
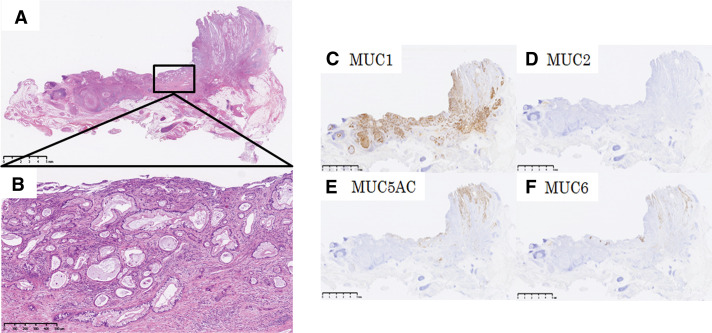
Microscopic analysis and MUC staining. Microscopically, the high columnar epithelium with papillary growth infiltrates into the stroma and submucosa of the common bile duct (**A**, **B**). MUC staining of the resected specimen showing positive expression for MUC 1, negative for MUC2, and positive for MUC5AC and MUC6 on the surface of the tumor (**C**–**F**). MUC, Mucin

Endoscopic retrograde cholangiography and subsequent intraductal ultrasonography showed that the tumor was located between the CHD and distal bile duct and extended into the intrapancreatic duct (**Fig. 2**). Subsequently, a drainage tube was inserted into the CBD to improve jaundice. After brush cytology of the CBD revealed strong cellular atypia and suspected malignancy, the patient was diagnosed with Bismuth type 1 perihilar cholangiocarcinoma. Therefore, subtotal stomach-preserving pancreaticoduodenectomy was performed. During the laparotomy, no liver metastasis or peritoneal dissemination was observed. The hepatoduodenal ligament exhibited severe adhesions around the duodenum from the initial surgery. A frozen section of the cut edge of the CHD showed negative finding for malignancy; therefore, we performed a subtotal stomach-preserving pancreaticoduodenectomy with modified Child’s reconstruction and regional lymphadenectomy. The operative time was 376 minutes, and estimated blood loss was 283 mL. Macroscopic examination of the surgical specimen revealed a 5-mm papillary nodule in the cystic duct (**Fig. 3**). Microscopic examination indicated that high columnar epithelium with papillary growth proliferated and infiltrated into the stroma and submucosa of the CBD (**Fig. 4A**, **4B**). High-grade dysplasia was observed in the CBD (**Fig. 3B**, **3C**).

**Fig. 5:**
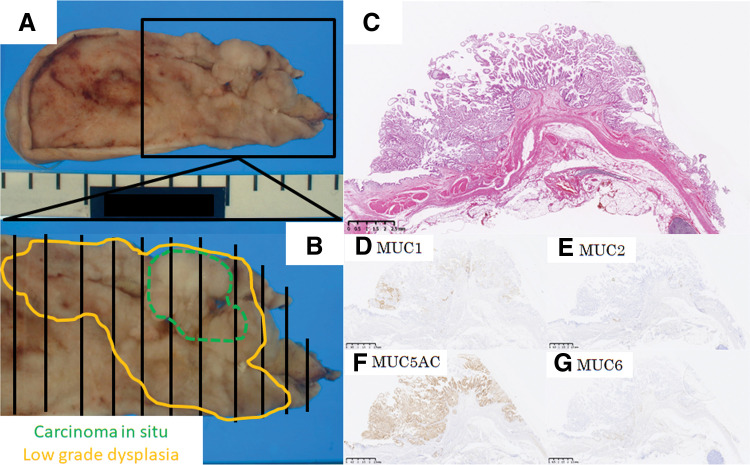
Histological analysis and MUC staining of the previously resected gallbladder tumor. Specimen of the gallbladder resected 19 years earlier showing a papillary tumor without invasion (**A**–**C**). Green dotted line and yellow line represent the areas of carcinoma *in situ* and low-grade dysplasia, respectively (**B**). MUC staining demonstrating focally positive finding for MUC1, negative finding for MUC2 and MUC6, and positive finding for MUC5AC (**D**–**G**). MUC, Mucin

Immunohistochemical analysis confirmed that the tumor cells were positive for MUC1, positive for MUC5AC and MUC6 on the surface, and negative for MUC2 (**Fig. 4C**–**4F**). Thus, the patient was diagnosed with pancreatobiliary-type ICPN associated with invasive carcinoma, 30 × 25 mm, G2-3, papillary carcinoma, invasive, pT3 pN1 M0, pStage IIIB. The cut edge of the bile duct showed negative finding for neoplastic cells. A retrospective review of the gallbladder specimen resected 19 years ago helped identify a papillary tumor with carcinoma in situ and low-grade dysplasia. MUC staining profiles showed focally positive findings for MUC1, negative findings for MUC2 and MUC6, and positive findings for MUC5AC (**Fig. 5A**–**5G**). The resection margin was located at the edge of cystic duct of gallbladder side, and the cut edge showed neither malignancy nor dysplasia. The distance from the tumor to the cystic duct stump was 6 mm. Although the concept of ICPN was not established 19 years ago, the tumor was diagnosed as ICPN under current classifications. Given the similarity in both the pathological morphology and MUC staining between the gallbladder lesion from 19 years ago and the current cystic duct lesion, the patient was diagnosed with metachronous occurrence of ICPN.

Although the patient developed a pancreatic fistula (International Study Group of Pancreatic Fistula grade B), we removed the drainage tube on postoperative day 24. He was discharged on postoperative day 26. At the time of this report, the patient has remained well and showed no signs of recurrence 6 months postoperatively.

## DISCUSSION

In 2012, Adsay et al. defined ICPN as gallbladder papillary neoplasms characterized by being intramucosal, pre-invasive, mass-forming, exophytic (either papillary or polypoid), at least 1.0 cm in size, compact, and distinct from the neighboring mucosa.^2)^ The 2019 World Health Organization classification of digestive system tumors recognizes ICPN as a unique pre-invasive neoplasm of the gallbladder, with lesions containing invasive carcinoma components classified under ICPN with associated carcinoma.^7)^ ICPN shares clinicopathological features with IPMN and IPNB and is therefore acknowledged as a counterpart to these conditions. While metachronous occurrences in the remnant pancreatobiliary tract have been documented for IPMN and IPNB,^3,4,8–10)^ such reports are rare in ICPN. Although several cases of metachronous biliary tract papillary carcinoma following resection of primary gallbladder lesions have been reported,^11–15)^ these cases are located in the common bile duct. Therefore, to the best of our knowledge, we present the first surgical report of metachronous ICPN in a remnant cystic duct.

Both IPMN and IPNB can develop multiple synchronous or metachronous lesions, with the following three possible mechanisms for the postoperative development of secondary lesions: metastatic or local recurrence of the primary lesion, metachronous secondary occurrence of new lesions, and progression of untreated lesions.^5,6,8–10)^ In our patient, the resected margin of the cystic duct during the initial surgery was malignancy- and dysplasia-free; however, secondary lesions developed 19 years post-cholecystectomy. Although determining the precise origin of the secondary tumor is challenging, these findings suggest a metachronous secondary occurrence of ICPN.

The prognosis for ICPN after resection is reportedly more favorable than that of conventional gallbladder carcinoma, with a 5-year overall survival of 78% for non-invasive ICPN, 60% for ICPN associated with carcinoma, and 18%–30% for conventional gallbladder carcinoma.^2)^ Although the treatment strategy for recurrent ICPN in the biliary tract remains unclear, complete surgical resection is believed to yield favorable outcomes, as similarly reported for recurrent IPMN^16)^ and IPNB.^17)^ Given the negative margins at the initial surgery, we believe further resection was unnecessary at that time. However, complete resection of the biliary tree during the initial surgery might have prevented the metachronous occurrence of ICPN.

The natural history of ICPN remains to be fully elucidated. Although some studies report that ICPN progression could span several years,^18,19)^ others have observed progression or recurrence within months.^20,21)^ In our case, the development of a secondary lesion occurred over 19 years, supporting the “field defect” theory advocated in IPMN, which suggests that all pancreatic ductal epithelial cells harbor the risk of dysplasia, and therefore, the remnant pancreas after partial pancreatectomy remains susceptible to developing significant lesions.^22)^

European guidelines and revised international guidelines for managing IPMN recommend lifetime surveillance for patients with IPMN post-pancreatectomy, provided the patients are fit for surgery.^23,24)^ As ICPN is considered the counterpart of IPMN, follow-up of the remnant biliary system is recommended, even after surgery for the primary lesion, through lifelong surveillance every 6 months during the early postoperative period and at least once a year in the late postoperative period.^25)^

## CONCLUSIONS

Our case involving a patient who had a secondary ICPN lesion 19 years post-initial surgery highlights the imperative for persistent surveillance. Given the incompletely understood natural history of ICPN, thorough long-term monitoring is crucial to effectively detect and manage potential recurrences.

## ACKNOWLEDGMENTS

We would like to thank Editage (www.editage.com) for the English language editing.

## DECLARATIONS

### Funding

The authors declare that this work was not supported by any grants or funding.

### Authors’ contributions

All authors have read and approved the manuscript.

H.K. drafted the manuscript. K.D. and M.T. supervised this study.

Y.K., T.A., S.Y., E.I., K.Shig., K.Shim., T.T., R.I., H.N., K.K., G.H., and Y.S. performed perioperative patient management.

### Availability of data and materials

Not applicable.

### Ethics approval and consent to participate

Ethical approval was obtained from the Ethics Committee of St. Mary’s Hospital (Approval No.: 24-1103).

### Consent for publication

Written informed consent was obtained from the patient for the publication of this report and its accompanying images.

### Competing interests

The authors declare that they have no competing interests.
